# Long-term outcomes in patients who received veno-venous extracorporeal membrane oxygenation and renal replacement therapy: a retrospective cohort study

**DOI:** 10.1186/s13613-022-01046-0

**Published:** 2022-07-23

**Authors:** Nuttha Lumlertgul, Rebeka Wright, Gareth Hutson, Jovana Kusic Milicevic, Georgios Vlachopanos, Ken Cheah Hooi Lee, Leah Pirondini, John Gregson, Barnaby Sanderson, Richard Leach, Luigi Camporota, Nicholas A. Barrett, Marlies Ostermann

**Affiliations:** 1 Department of Critical Care, Guy’s & St Thomas’ Hospital, NHS Foundation Trust, 249 Westminster Bridge Road, London, UK; 2 Division of Nephrology and Excellence Centre for Critical Care Nephrology, King Chulalongkorn Memorial Hospital, Bangkok, Thailand; 3 Centre of Excellence in Critical Care Nephrology, Chulalongkorn University, Bangkok, Thailand; 4 Department of Nephrology, Clinical Hospital Centre Zemun, Belgrade, Serbia; 5 Department of Nephrology, General Hospital of Nikea, Athens, Greece; 6 Department of Respiratory and Critical Care Medicine, Singapore General Hospital, Singapore, Singapore; 7 Department of Medical Statistics, London School of Hygiene and Tropical Medicine, London, UK; 8 Department of Critical Care, King’s College, Guy’s & St Thomas’ NHS Foundation Trust, London, SE1 7EH UK

**Keywords:** Acute kidney injury, Chronic kidney disease, ECMO, Extracorporeal membrane oxygenation, Kidney replacement therapy, Mortality, Renal replacement therapy

## Abstract

**Background:**

Acute kidney injury (AKI) is a frequent complication in patients with severe respiratory failure receiving extracorporeal membrane oxygenation (ECMO). However, little is known of long-term kidney function in ECMO survivors. We aimed to assess the long-term mortality and kidney outcomes in adult patients treated with veno-venous ECMO (VV-ECMO).

**Methods:**

This was a single-centre retrospective study of adult patients (≥ 18 years old) who were treated with VV-ECMO at a commissioned ECMO centre in the UK between 1st September 2010, and 30th November 2016. AKI was defined and staged using the serum creatinine and urine output criteria of the Kidney Diseases: Improving Global Outcomes (KDIGO) classification. The primary outcome was 1-year mortality. Secondary outcomes were long-term mortality (up to March 2020), 1-year incidence of end-stage kidney disease (ESKD) or chronic kidney disease (CKD) among AKI patients who received renal replacement therapy (AKI-RRT), AKI patients who did not receive RRT (AKI-no RRT) and patients without AKI (non-AKI).

**Results:**

A total of 300 patients [57% male; median age 44.5; interquartile range (IQR) 34–54] were included in the final analysis. Past medical histories included diabetes (12%), hypertension (17%), and CKD (2.3%). The main cause of severe respiratory failure was pulmonary infection (72%). AKI occurred in 230 patients (76.7%) and 59.3% received renal replacement therapy (RRT). One-year mortality was 32% in AKI-RRT patients vs. 21.4% in non-AKI patients (*p* = 0.014). The median follow-up time was 4.35 years. Patients who received RRT had a higher risk of 1-year mortality than those who did not receive RRT (adjusted HR 1.80, 95% CI 1.06, 3.06; *p* = 0.029). ESKD occurred in 3 patients, all of whom were in the AKI-RRT group. At 1-year, 41.2% of survivors had serum creatinine results available. Among these, CKD was prevalent in 33.3% of AKI-RRT patients vs. 4.3% in non-AKI patients (*p* = 0.004).

**Conclusions:**

VV-EMCO patients with AKI-RRT had high long-term mortality. Monitoring of kidney function after hospital discharge was poor. In patients with follow-up creatinine results available, the CKD prevalence was high at 1 year, especially in AKI-RRT patients. More awareness about this serious long-term complication and appropriate follow-up interventions are required.

**Supplementary Information:**

The online version contains supplementary material available at 10.1186/s13613-022-01046-0.

## Introduction

Extracorporeal membrane oxygenation (ECMO) is a rescue therapy in patients with severe respiratory failure, refractory cardiogenic shock, or combined cardiorespiratory failure. Two landmark randomised controlled trials and a subsequent individual-patient meta-analysis have suggested survival benefits and less organ failure in patients with severe acute respiratory distress syndrome (ARDS) treated with veno-venous ECMO (VV-ECMO) compared to conventional management [1–3]. The use of ECMO in adult patients with refractory respiratory failure has risen significantly in the past decade, including during the coronavirus-19 (COVID-19) pandemic [4–6].

Acute kidney injury (AKI) is a common complication in patients receiving ECMO [7]. The reported AKI incidence ranges from 26 to 85%. Overall, 40–60% of ECMO patients receive renal replacement therapy (RRT), but clinical practice varies [7, 8]. The pathophysiology of AKI is multifactorial and includes patient-related factors (e.g. pre-existing comorbidities, haemodynamics, exposure to nephrotoxic agents, severe hypoxemia, hypercapnia, systemic inflammation, neurohormonal dysregulation), the impact of mechanical ventilation on kidney function and ECMO-related factors (e.g. ischaemia–reperfusion injury, continuous flow during veno-arterial (VA-) ECMO, haemolysis, bioincompatibility and hypercoagulability) [9].

Short-term outcomes are worse in patients who develop AKI during ECMO compared to patients without AKI, including higher risk of in-hospital mortality, sepsis, fluid overload, bleeding, and failure to wean from ECMO [10, 11]. However, little is known about the long-term kidney outcomes in ECMO survivors. This question is particularly important since research in non-ECMO patients has highlighted the high risk of chronic kidney disease (CKD) and end-stage kidney disease (ESKD) in AKI survivors and the negative impact of lack of follow-up [12, 13].

The aim of this study was to determine the short and long-term patient and kidney outcomes of patients who received VV-ECMO. In addition, we aimed to explore the correlation between serum creatinine results at hospital discharge, 6 months, and 1 year.

## Material and methods

### Setting

Guy’s & St Thomas’ Hospital is one of 6 commissioned ECMO centres in the UK National Health Service (NHS). The ECMO service was set up in 2010 and serves a sixth of the country with a catchment area of around 17 million people [14].

### Study design and participants

We retrospectively identified all patients who received VV-ECMO at our centre between September 1, 2010, and November 30, 2016. VV-ECMO was initiated in patients with severe acute respiratory failure refractory to conventional management as specified in the ECMO commissioning criteria, with the ultimate decision at the discretion of the clinical team [15] (Additional file 2). Exclusion criteria were: (1) age younger than 18 years; (2) receipt of VA-ECMO or extracorporeal carbon dioxide removal (ECCO_2_R); (3) death within 48 h of ECMO initiation; (4) pre-existing ESKD, and (5) history of kidney transplantation.

### Data collection

Data were extracted from electronic health records by trained researchers. The following variables on admission were collected: demographic data, comorbidities, Sequential Organ Failure Assessment (SOFA) score, laboratory parameters, details about ECMO (indications, timing, initial settings, duration), urine output, and fluid balance. AKI was defined and staged according to the Kidney Disease: Improving Global Outcomes (KDIGO) classification using both serum creatinine (SCr) and urine output criteria [16]. Baseline kidney function was defined as the most recent outpatient non-emergency SCr concentration between 7 and 365 days before admission. In patients with missing baseline SCr results, we followed the KDIGO guideline and used the first SCr on day of hospital admission or performed back calculation of SCr based on an estimated glomerular filtration rate (GFR) of 75 mL/min/1.73 m^2^ using the modification of diet in renal disease (MDRD) formula [16]. The following details for AKI were collected: criteria, onset, duration and RRT requirement including onset relative to ICU admission and ECMO initiation.

We also collected data related to in-hospital complications, e.g. sepsis, bleeding, thrombosis, stroke, and units of blood product transfusion. For in-hospital outcomes, we recorded the duration of ICU and hospital stay and duration of mechanical ventilation, RRT, and ECMO support. In hospital survivors, we obtained survival data through NHS records and the incidence of new-onset ESKD from the United Kingdom Renal Registry (UKRR), a mandatory ESKD registry in the UK. SCr values after discharge at 6 months and 12 months (or the closest values within 3 months) were acquired from local health records or by contacting general practitioners (GPs) via phone calls or emails. CKD was defined as persistent GFR < 60 mL/min/1.73 m^2^ for > 3 months and staged according to the CKD Epidemiology Collaboration (CKD-EPI) equation [17].

### Outcomes

The primary outcome was 1-year mortality. We differentiated between patients with AKI who received RRT (AKI-RRT), patients with AKI not treated with RRT (AKI-no RRT) and patients without AKI (non-AKI). Secondary outcomes were: (1) long-term mortality (up to March 2020); (2) the incidence of CKD and ESKD at 1 year among AKI-RRT, AKI-no RRT, and non-AKI patients; (3) SCr results at 6 months and 1 year and (4) changes and correlation of SCr results at hospital discharge, 6 months, and at 12 months. The last follow-up date for survival status was 4th March 2020, the date of the last available data from the NHS records at the time of the study end.

### Statistical analysis

Baseline characteristics were summarised by AKI diagnosis and RRT status. Continuous variables were reported as median (interquartile range, IQR) and categorical variables as number (percentage). Hospital and 1-year outcomes were tabulated by AKI and RRT status. We analysed long-term mortality from the date of ECMO initiation according to AKI and RRT status using Kaplan–Meier curves, and by fitting a Cox proportional hazards model with AKI and RRT status as a time-updated covariate. To identify and adjust for relevant confounders associated with mortality, we used Cox proportional hazards modelling with a forward stepwise selection algorithm. We used a *p*-value threshold of 0.05 for inclusion and forced age at admission into the model as a covariate that was a priori likely to be associated with mortality. Since a proportion of patients did not have SCr results available at 1 year, we compared characteristics of one-year survivors with and without follow-up SCr measurements. We examined Spearman’s correlations between creatinine measurements at discharge and at 6 and 12 months following discharge. Analyses were conducted with R version 3.6.1.

## Results

### Baseline characteristics

Between 1st September 2010 and 30th November 2016, 346 patients were treated with ECMO at Guy’s & St Thomas’ NHS Foundation Trust. Following exclusion of 46 patients, 300 were included in the final analysis (Additional file 1: Fig. S1). Their median age was 44.5 (interquartile range [IQR] 34–54)], 57% were male, and the median body mass index (BMI) was 27.3 (IQR 24.1–33.4) kg/m^2^. Past medical histories included diabetes (12%), hypertension (17%), and pre-existing CKD (2.3%). The median SOFA score on admission to ICU was 9 (IQR 6–12). The main indication for VV-ECMO was respiratory failure due to infection (72%) (Table 1). The total number of patients who received ECMO by year is shown in Additional file 1: Table S1. The majority of the patients were initiated on ECMO on the same day or within 1 day of ICU admission (Additional file 1: Table S2). The median duration of ECMO treatment was 9 days (IQR 6–16 days) and median length of hospital stay was 26 days (IQR 17–45 days).

**Table 1:** Baseline characteristics of patients by AKI and RRT status

Characteristic^a^	Overall, *N* = 300^a^	AKI, RRT, *N* = 178	AKI, no RRT, *N* = 52	No AKI, *N* = 70	*P*-value (all groups)^b^	*P*-value (AKI vs. no AKI)^c^
Sex					0.010	0.003
Male	171 (57%)	109 (61%)	33 (63%)	29 (41%)		
Female	129 (43%)	69 (39%)	19 (37%)	41 (59%)		
Ethnicity^d^					0.312	0.121
White	230 (77%)	138 (78%)	38 (73%)	54 (78%)		
Black	29 (9.8%)	14 (8.0%)	5 (9.6%)	10 (14%)		
Asian	27 (9.1%)	16 (9.1%)	6 (12%)	5 (7.2%)		
Mixed	0 (0%)	0 (0%)	0 (0%)	0 (0%)		
Other/not-stated	11 (3.7%)	8 (4.5%)	3 (5.8%)	0 (0%)		
Age (years)	44.5 (34.0, 54.2)	48.5 (40.0, 57.0)	39.0 (30.8, 48.0)	41.0 (25.2, 48.0)	< 0.001	< 0.001
Weight (kg)^d^	80.0 (70.0, 100.0)	84.6 (70.0, 102.0)	80.0 (70.0, 102.0)	73.0 (60.0, 85.0)	0.002	< 0.001
BMI (kg/m^2^)^d^	27.3 (24.1, 33.4)	27.7 (24.4, 33.8)	28.6 (24.5, 32.6)	25.4 (22.0, 31.1)	0.039	0.013
Diabetes	37 (12%)	26 (15%)	6 (12%)	5 (7.1%)	0.269	0.131
Hypertension	52 (17%)	39 (22%)	5 (9.6%)	8 (11%)	0.039	0.136
Congestive heart failure	8 (2.7%)	6 (3.4%)	1 (1.9%)	1 (1.4%)	0.881	0.686
Coronary artery disease	8 (2.7%)	5 (2.8%)	1 (1.9%)	2 (2.9%)	> 0.999	> 0.999
Atrial fibrillation	4 (1.3%)	3 (1.7%)	0 (0%)	1 (1.4%)	> 0.999	> 0.999
Peripheral artery disease	1 (0.3%)	1 (0.6%)	0 (0%)	0 (0%)	> 0.999	> 0.999
Cerebrovascular accidents (history)	6 (2.0%)	3 (1.7%)	2 (3.8%)	1 (1.4%)	0.523	> 0.999
Chronic lung disease	71 (24%)	34 (19%)	13 (25%)	24 (34%)	0.039	0.017
Chronic liver disease	10 (3.3%)	9 (5.1%)	1 (1.9%)	0 (0%)	0.109	0.124
Any active malignancy within 5 yrs	14 (4.7%)	9 (5.1%)	2 (3.8%)	3 (4.3%)	> 0.999	> 0.999
Type of malignancy					> 0.999	> 0.999
Solid tumour	5 (1.7%)	3 (1.7%)	1 (1.9%)	1 (1.4%)		
Haematologic tumour	9 (3.0%)	6 (3.4%)	1 (1.9%)	2 (2.9%)		
Other immunosuppressive conditions	30 (10%)	22 (12%)	2 (3.8%)	6 (8.6%)	0.178	0.649
Chronic kidney disease (history)	7 (2.3%)	6 (3.4%)	1 (1.9%)	0 (0%)	0.308	0.206
Use of nephrotoxic drugs^e^	78 (26%)	56 (31%)	8 (15%)	14 (20%)	0.029	0.191
SOFA score on day 1 of ICU admission	9.0 (6.0, 12.0)	11.0 (8.0, 14.0)	8.0 (5.0, 9.0)	7.0 (4.0, 8.0)	< 0.001	< 0.001
Reason for ECMO support					*(groups too small)*	0.002
Infection	215 (72%)	143 (80%)	33 (63%)	39 (56%)		
Immune/inflammation	24 (8.0%)	9 (5.1%)	5 (9.6%)	10 (14%)		
Asthma or COPD exacerbation	20 (6.7%)	2 (1.1%)	7 (13%)	11 (16%)		
Trauma	12 (4.0%)	7 (3.9%)	2 (3.8%)	3 (4.3%)		
Postoperative	2 (0.7%)	1 (0.6%)	1 (1.9%)	0 (0%)		
Cardiac	8 (2.7%)	6 (3.4%)	1 (1.9%)	1 (1.4%)		
Others	19 (6.3%)	10 (5.6%)	3 (5.8%)	6 (8.6%)		
ECMO speed (rpm)—initial setting	3,132.5 (2820.0, 3496.2)	3,202.5 (2832.5, 3500.0)	3,145.0 (2828.8, 3586.2)	3,017.5 (2761.2, 3330.0)	0.134	0.047
ECMO duration (days)	9.0 (6.0, 16.0)	10.0 (7.0, 16.0)	8.0 (6.0, 14.0)	8.0 (6.0, 17.8)	0.131	0.389
Baseline serum creatinine (µmol/L)	80 (69, 97)	93 (76, 99)	82 (68, 98)	60 (47, 74)	0.0001	< 0.001

*AKI* acute kidney injury, *BMI* body mass index, *COPD* chronic obstructive lung disease, *ECMO* extracorporeal membrane oxygenation, *ICU* intensive care unit, *SOFA* Sequential Organ Failure Assessment, *RRT* renal replacement therapy

^a^*n* (%); median (IQR)

^b^Categorical variables: Pearson’s Chi-squared test or Fisher’s exact test for small group sizes; continuous variables: Kruskal–Wallis rank sum test

^c^Categorical variables: Pearson’s Chi-squared test or Fisher’s exact test for small group sizes; continuous variables: Wilcoxon rank sum test

^d^Missing data: ethnicity (*n* = 3), weight (*n* = 15), BMI (*n* = 45)

^e^Defined as the receipt of non-steroidal anti-inflammatory drugs, contrast media or nephrotoxic antibiotics (e.g. aminoglycosides, vancomycin, piperacillin–tazobactam, colistin) within 7 days prior to ECMO initiation

### AKI diagnosis and RRT

True baseline SCr values were available in 33 (11%). AKI was diagnosed in 230 patients (76.7%) of whom 178 (59.3% of all patients) received RRT. The AKI diagnosis was based on SCr results in 129 (56.1%), urine output in 53 (23.0%), and both criteria in 48 (20.9%) of all AKI patients. AKI was diagnosed before ECMO initiation in 25.6% and on the same day or after ECMO start date in 74.4%, respectively. RRT was started before ECMO initiation in 17.4% and on the same day or after ECMO cannulation in 82.6% (Additional file 1: Tables S3, S4). Patients in the AKI-RRT group were older, had higher BMI and baseline SCr values, had more hypertension and higher SOFA score on admission, and were more likely to be exposed to nephrotoxic drugs than those in the non-AKI and AKI-no RRT groups (Table 1). The main indications for RRT initiation were volume control/fluid overload, followed by oliguria, uraemia then acidosis (Additional file 1: Table S5).

### In-hospital outcomes

The in-hospital mortality was 27.0% in AKI-RRT patients compared with 11.5% in the AKI-no RRT and 15.7% in non-AKI cohorts. Patients with AKI-RRT had a longer ICU stay and needed mechanical ventilation and ECMO for longer durations. They were also more likely to have complications, including sepsis, bleeding, stroke or thrombosis, received more blood products, and were less likely to be liberated from ECMO (Table 2). In AKI-RRT patients, the median RRT duration was 14 days (IQR 9–24) while the median ECMO duration was 10 days (IQR 7–16). At ICU discharge, 45 of 235 ICU survivors (19.1%) were still RRT-dependent. At hospital discharge, 7 of 212 hospital survivors (3.3%) were RRT-dependent.

**Table 2:** In-hospital outcomes by acute kidney injury and renal replacement therapy status

Characteristic^a^	Overall, *N* = 300	AKI, RRT, *N* = 178	AKI, no RRT, *N* = 52	No AKI, *N* = 70
In-hospital mortality	65 (21.7%)	48 (27.0%)	6 (11.5%)	11 (15.7%)
ICU length of stay (days)	21 (14, 32)	24 (16, 38)	17 (11, 26)	19 (12, 32)
Hospital length of stay (days)	26 (17, 45)	27 (18, 47)	24 (15, 35)	28 (16, 46)
ECMO duration (days)	9 (6, 16)	10 (7, 16)	8 (6, 14)	8 (6, 18)
ECMO weaning	240 (80.0%)	133 (74.7%)	47 (90.4%)	60 (85.7%)
MV duration (days)	16 (10, 26)	18 (12, 29)	14 (9, 18)	12 (7, 25)
Sepsis	157 (52.3%)	102 (57.3%)	24 (46.2%)	31 (44.3%)
Bleeding	66 (22.0%)	49 (27.5%)	6 (11.5%)	11 (15.7%)
Stroke	42 (14.0%)	7 (13.5%)	30 (16.9%)	5 (7.1%)
Thrombosis	84 (28.0%)	53 (29.8%)	13 (25.0%)	18 (25.7%)
Total number of packed red blood cell units during ICU admission (*n* = 256)^b^	6 (3, 12)	9 (5, 17)	3 (2, 5)	4 (2, 8)
Total number of cryoprecipitate/FFP units during ICU admission (*n* = 86)^b^	6 (4, 13)	8 (4, 15)	2 (2, 6)	6 (4, 7)
Total number of platelets during ICU admission (*n* = 127)^b^	5 (3, 10)	5 (4, 11)	1 (1, 2)	4 (2, 6)

*AKI* acute kidney injury, *ECMO* extracorporeal membrane oxygenation, *FFP* fresh frozen plasma, *ICU* intensive care unit, *MV* mechanical ventilation, *RRT* renal replacement therapy

^a^Statistics presented: *n* (%), median (interquartile range)

^b^Excluded those who did not receive blood products during admission

### One-year and long-term mortality

The overall 1-year mortality was 32.0% in AKI-RRT patients compared with 13.5% in the AKI-no RRT and 21.4% in non-AKI cohorts (*p* = 0.014 for AKI-RRT vs. no-AKI; unadjusted HR 2.01, 95% CI 1.15–3.51 and *p* = 0.241 for AKI-no RRT vs. no-AKI; unadjusted HR 0.57, 95% CI 0.22–1.46) (Table 3). Univariate Cox regression analysis showed that AKI-RRT, age, chronic liver disease, malignancy, low baseline serum albumin, and SOFA score on admission were associated with 1-year mortality. Multivariate analysis demonstrated that patients who received RRT had a 1.80-fold (95% CI 1.06–3.06, *p* = 0.029) higher hazard of 1-year mortality than those who did not receive RRT (Table 4).

**Table 3:** Primary and secondary outcomes at 1 year by acute kidney injury and renal replacement therapy status

Characteristic^a^	Overall *N* = 300	AKI, RRT *N* = 178	AKI, no RRT *N* = 52	No AKI *N* = 70	*P*-value^b^ (AKI, no RRT vs. no AKI)	*P*-value^b^ (AKI, RRT vs. no AKI)
1-year mortality (%)	79 (26.3%) 95% CI 21.4–31.7%	57 (32.0%) 95% CI 25.2–39.4%	7 (13.5%) 95% CI 5.6–25.8%	15 (21.4%) 95% CI 12.5–32.9%	0.241	0.014
(Survivors)	221	121	45	55	–	–
30-day mortality (%)	54 (18.0%) 95% CI 13.8–22.8%	41 (23.0%) 95% CI 17.1–29.9%	6 (11.5%) 95% CI 4.4–23.4%	7 (10.0%) 95% CI 4.1–19.5%	0.974	0.003
End-stage kidney disease (% among survivors)	3 (1.4%) 95% CI 0.3–3.9%	3 (2.5%) 95% CI 0.5–7.1%	0 (0.0%) 95% CI 0.0–7.9%	0 (0.0%) 95% CI 0.0–6.5%	NA	0.561
Mean (SD) serum creatinine in µmol/L^c^						
6 months	85.6 (40.9)	90.8 (49.3)	86.6 (16.9)	72.4 (19.9)	0.060	0.161
1 year	89.4 (53.6)	99.4 (69.3)	81.0 (18.5)	74.7 (15.7)	0.368	0.123
1 year, or 6 months if unavailable	89.7 (51.7)	98.4 (64.0)	85.3 (18.5)	71.8 (16.6)	0.033	0.026
Chronic kidney disease at 1 year (% among survivors with SCr available)^d^	19 (20.9%) 95% CI 13.1–30.7%	18 (33.3%) 95% CI 21.1–47.5%	0 (0.0%) 95% CI 0.0–23.2%	1 (4.3%) 95% CI 0.1–21.9%	> 0.999	0.004

*AKI* acute kidney injury, *ICU* intensive care unit, *RRT* renal replacement therapy, *SCr* serum creatinine, *NA* not available

^a^Statistics presented: *n* (%)

^b^*P*-values are from a Cox proportional hazard model with AKI-RRT status as a time-dependent covariate for mortality outcomes, and Fisher’s exact test for ESKD and CKD outcomes

^c^Available in 80 patients at 6 months, 65 patients at 12 months and 91 patients at either 6 or 12 months

^d^Among 91 patients with a 6 or 12 month SCr measurement

**Table 4:** Unadjusted and adjusted hazard ratios of the association between clinical characteristics and 1-year mortality

Characteristic	Univariate analysis			Multivariable model^a^		
	Hazard ratio	95% CI	*P*-value	Adjusted hazard ratio	95% CI	*P*-value
AKI/RRT group						
No AKI	1 (reference)	–	–	1 (reference)		
AKI without RRT	0.57	0.22, 1.46	0.241			
AKI with RRT	2.01	1.15, 3.51	0.014	1.80	1.06, 3.06	0.029
Age (per 5 years)	1.10	1.01, 1.19	0.022	1.06	0.98, 1.15	0.171
Female sex	1.00	0.64, 1.56	0.988			
BMI (per kg/m^2^)	0.98	0.95, 1.02	0.335			
Chronic liver disease	3.71	1.61, 8.55	0.002	4.36	1.83, 10.4	< 0.001
Malignancy	5.98	3.15, 11.4	< 0.001	7.68	3.95, 14.9	< 0.001
SOFA score on admission	1.08	1.02, 1.14	0.009*			
Diabetes	1.41	0.78, 2.56	0.253			
Hypertension	1.28	0.74, 2.21	0.384			
Congestive heart failure	0.41	0.06, 2.92	0.371			
CKD	0.50	0.07, 3.56	0.485			
Coronary artery disease	1.42	0.45, 4.50	0.551			
Atrial fibrillation	1.90	0.47,7.73	0.371			
Cerebrovascular accidents	1.23	0.30, 5.02	0.770			
Chronic lung disease	0.71	0.40, 1.25	0.232			
Infectious vs. non-infectious aetiology	0.85	0.53, 1.37	0.503	0.62	0.38, 1.01	0.054
Baseline serum albumin on admission (per 10 g/L)	0.57	0.35, 0.91	0.018	0.55	0.32, 0.95	0.031

*RRT* renal replacement therapy, *BMI* body mass index, *SOFA* Sequential Organ Failure Assessment, *CKD* chronic kidney disease, *ECMO* extracorporeal membrane oxygenation, *HR* hazard ratio, *CI* confidence interval

*SOFA score not selected for multivariable analysis due to the collinearity with RRT status

^a^Multivariable model includes RRT status (as a time-dependent covariate), age, chronic liver disease, malignancy, infectious vs. non-infectious aetiology and baseline serum albumin

**Fig. 1:**
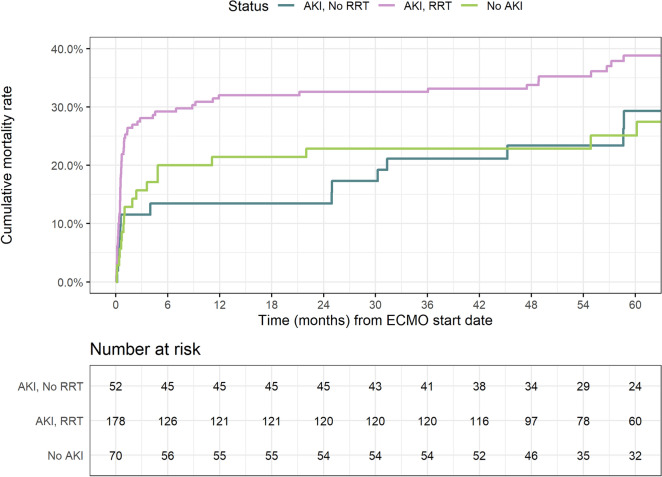
Kaplan–Meier estimates of long-term mortality according to acute kidney injury (AKI) and renal replacement therapy (RRT) status

The median follow-up time was 4.35 years. Kaplan–Meier estimates of overall mortality according to AKI and RRT status are shown in Fig. 1. Patients who received RRT had higher risk of overall mortality than those who did not receive RRT (unadjusted HR 1.70, 95% CI 1.13, 2.55; *p* = 0.011). The higher mortality among AKI-RRT patients was mainly driven by a high mortality rate early in follow-up (Fig. 1). Among RRT patients, 1-year mortality rate was higher in those in whom RRT was initiated on the same day or after ECMO cannulation (35.2% and 32.2%) than in patients in whom RRT was started before ECMO (22.6%) (Additional file 1: Table S6) However, the adjusted HR by RRT initiation relative to ECMO start date did not show an association with overall mortality (Additional file 1: Table S7).

### One-year kidney outcomes

At 6 months, 5 of 227 6-month survivors (2.2%) were RRT-dependent. Three of 221 hospital survivors (1.4%) developed new ESKD by 1 year; all 3 patients had received RRT for AKI during admission. Among survivors, 91 (41.2%) patients had follow-up SCr measurements within 1 year after hospital discharge; 26 (11.8%) had only one result available at 6 months, 14 (6.3%) had only one result available at 12 months, and 51 (23.1%) had results available at both time points. Patients with SCr values available at 1 year were similar to patients without SCr results at 1 year in terms of baseline characteristics, SCr results at hospital admission and discharge, and receipt of RRT during hospitalisation (Additional file 1: Table S8).

Among 91 survivors with SCr results available at 6 or 12 months after discharge, SCr concentrations were highest in the AKI-RRT group (98.4 ± 64 µmol/L), followed by AKI-no RRT (85.3 ± 18.5 µmol/L) and no-AKI (71.8 ± 16.6 µmol/L) groups, respectively (*p* = 0.026 for AKI-RRT vs. no-AKI; *p* = 0.033 for AKI-no RRT vs. no-AKI) (Table 3).

Nineteen (20.9%) patients had prevalent CKD at 1 year; 33.9% in the AKI-RRT group compared with 4.3% in the non-AKI group (*p* = 0.004) (Table 3). The severity of CKD in the non-ESKD patients was CKD stage 3, 4, and 5 in 17, 1, and 1 patient, respectively. Patients in the AKI-RRT group who were still RRT-dependent at hospital discharge had a prevalence of CKD of 40.9% (9 of 22 patients with SCr available) at 1 year compared to 29.4% (10 of 34 patients with SCr available) in those who were liberated from RRT before discharge (*p* = 0.40).

### Correlation of SCr at discharge, 6 months, and 12 months

**Fig. 2:**
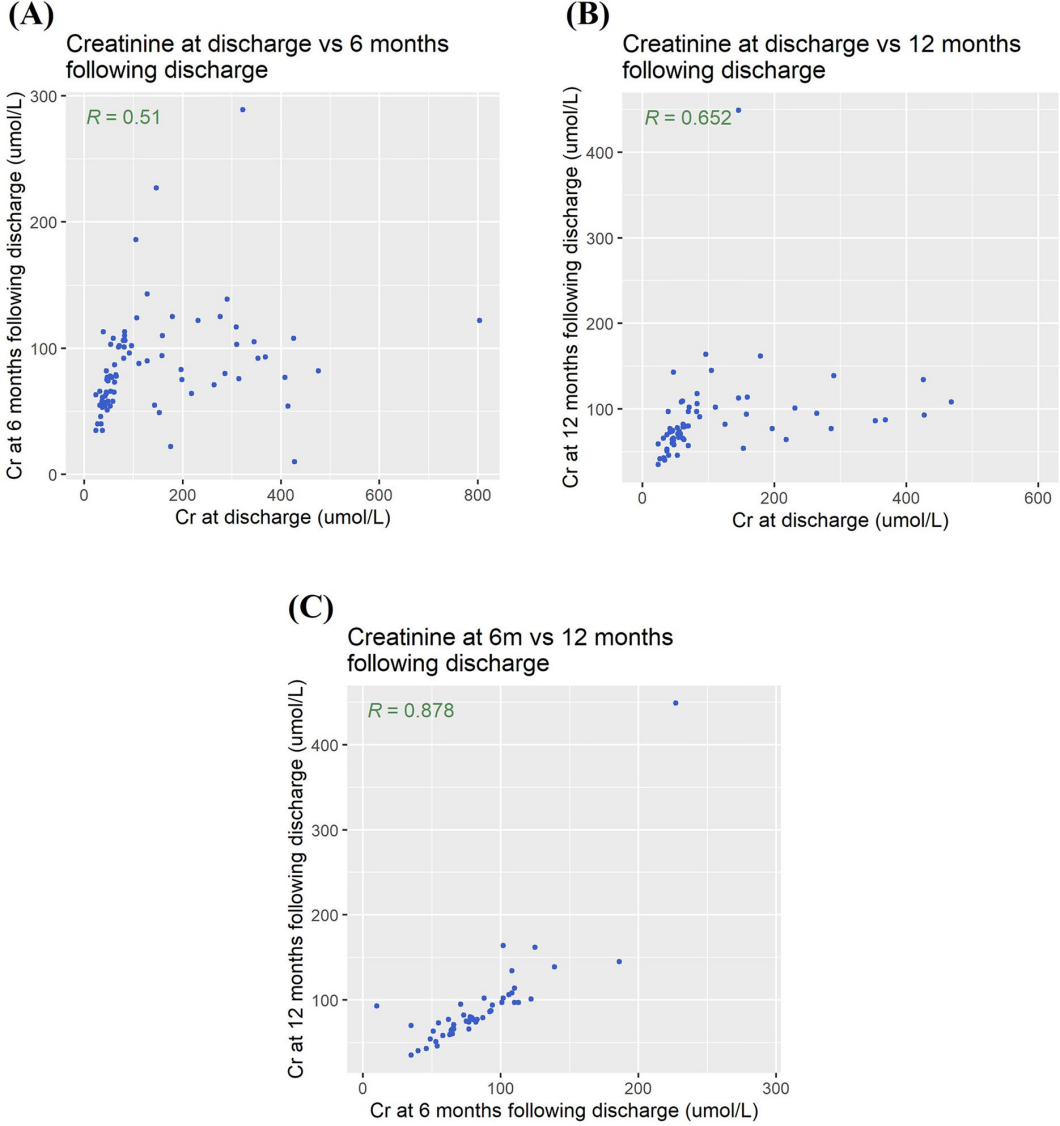
Scatter plots and Spearman’s rank correlation coefficients comparing creatinine at hospital discharge and at 6 months (*n* = 77) (**A**) and 12 months (*n* = 65) (**B**) following hospital discharge and between creatinine at 6 and 12 months (*n* = 51) (**C**), in patients who survived until 1 year with available creatinine measurements

In all patients with SCr results available at hospital discharge, 6 and 12 months (*n* = 51), the comparison of median SCr (IQR) is shown in Additional file 1: Fig. S2. SCr significantly increased from 70.0 (46.5, 169.0) µmol/L at discharge to 78.0 (61.0, 103.0) µmol/L at 6 months (*p* < 0.001), but there was no significant difference between the values at 6 and 12 months (*p* = 0.146). There was a modest correlation between SCr values at discharge and 6 months (*r* = 0.514, *p* < 0.001) and between SCr at discharge and 12 months (*r* = 0.652, *p* < 0.001). The relationship between SCr results at 6 and 12 months was stronger (*r* = 0.878, *p* < 0.001) (Fig. 2).

### Subgroup analysis

AKI incidence was underestimated when using first hospital admission SCr values while overestimated when using back-calculated SCr values. However, in-hospital and 1-year mortality rates were not different when comparing the 3 methods of determining baseline SCr values. A subgroup analysis of patients with true baseline SCr confirmed similar outcomes in the AKI-RRT group than the others (Additional file 1: Tables S9, S10).

## Discussion

This is the largest study exploring both short- and longer-term kidney outcomes in VV-ECMO patients. The key findings were: (i) AKI requiring RRT is common (59.3%); (ii) 19% were dialysis dependent at discharge; (iii) long-term mortality was highest in the AKI-RRT group; (iv) only 42% of survivors had follow-up SCr results available within 12 months after hospital discharge; (v) among patients with SCr results available, one-third of the AKI-RRT group had CKD compared with 4% in the non-AKI group, and (vi) SCr at discharge had a modest correlation with SCr at 6 or 12 months.

Despite a high ICU survival rate that correlates well with NHS England data [15], the incidence of AKI is high in patients receiving VV-ECMO. Current literature reports mortality rates of 55% in patients with severe AKI on VV-ECMO [18] which is much higher than in our patient cohort. This may be accounted for by various factors such as young age, high BMI, and the centre volume–outcome relationship [19, 20]. As in other studies in the literature, the outcomes of AKI-RRT patients were worse, including prolonged duration of mechanical ventilation and ECMO, longer length of ICU stay, higher incidence of sepsis, thrombosis, bleeding and stroke, need for more units of blood products, and increased mortality [10, 21, 22]. These findings might reflect a higher severity of illness. Other potential explanations are the negative impact of AKI on other organs, and the potential interactions between RRT and ECMO technologies [9]. However, the exact cause-and-effect relationship between in-hospital outcomes and AKI and RRT status was not established.

Long-term functional and psychological well-being of VV-ECMO survivors has been well described [23–25], but little data exist about their long-term kidney outcomes. Furthermore, in several previous studies, VA and VV ECMO patients were analysed together despite the fact that there are important differences in patient comorbidities as well as ECMO characteristics. In children receiving concomitant ECMO and continuous renal replacement therapy (CRRT), dialysis independence rates of 93% at discharge and 95% at 1 year have been reported [26, 27]. Two large cohort studies from the Taiwan’s National Health Insurance Research Database including VV- and VA-ECMO patients revealed increased risks of CKD, ESKD, and all-cause mortality in AKI-RRT patients (mean follow-up 2.4 ± 2.5 years per patient) [10, 28]. A study in VA-ECMO patients demonstrated 11% dialysis dependence (median follow-up 64 months in non-ESKD patients) and 51% prevalence of CKD at 1 year [29]. Another recent study of predominantly VA-ECMO survivors (80%) reported major adverse kidney events (median follow-up time 3.4 years) which include AKI, partial/no recovery at discharge, and RRT on the same date or after ECMO initiation [30]. In two studies of exclusively VV-ECMO patients, no and 6.8% patients were dialysis dependent at hospital discharge, but longer-term renal function was not reported [11, 31]. Our study includes only patients who received VV-ECMO for severe acute respiratory failure and focusses on long-term survival and kidney outcomes after discharge. Similar to previous literature, long-term mortality is highest in patients who received RRT [10, 28].

Although only 3 patients developed ESKD, it should be noted that they were young and had few comorbidities prior to the acute illness requiring ECMO. It is surprising and very concerning that less than 50% of patients had SCr results available within the first 12 months after discharge from hospital despite the fact that 60% of them had received RRT whilst on ECMO. One-third of the AKI-RRT group had CKD at 1 year. Although the CKD status could only be determined in patients with SCr results available, their baseline characteristics, RRT status during hospitalisation, and SCr values at discharge were comparable to those without follow-up SCr results. This leads us to speculate that CKD prevalence of the total cohort at 1 year may have indeed been in the range of 30%.

Identifying patients who are at high risk of long-term CKD after AKI is important. However, as in previous studies, we demonstrated that SCr at discharge was not predictive of subsequent SCr values [32, 33]. This may be the consequence of prolonged hospitalisation and the impact of muscle wasting, inflammation, sepsis, and malnutrition on creatinine generation [34]. Notably, previous studies reported sarcopenia in around 1 in 4 of patients who received ECMO on admission [35]. Consequently, GFR at hospital discharge can easily be overestimated leading to suboptimal follow-up and under-recognition of GFR decline. Over time, SCr returned to the steady state [32], hence the observed increase in SCr from discharge to 6 months and the improved correlation between SCr results at 6 and 12 months.

With increasing use of ECMO in acute respiratory failure and the associated high survival [15], attendance at ICU recovery clinics and general practice is expected to increase. Our study highlights the high incidence of CKD in ECMO survivors and the worrying lack of kidney function monitoring in this high-risk group. There is an urgent need for greater awareness and formal guidance to ensure appropriate and timely follow-up post-ECMO, in particular of patients who received RRT. As proposed for critically ill patients in general, AKI aftercare for ECMO patients requires a collaborative multidisciplinary effort by all teams involved, including intensivists, nephrologists, internists, GPs and pharmacists [36]. To facilitate this, comprehensive discharge documentation and information transfer are essential [37]. The hospital discharge summary should not only contain primary events such as AKI cause, staging and RRT receipt, but also recommendations for future management such as nephrotoxin avoidance, weight and blood pressure monitoring, cardiovascular medication management, and sick day rules. If possible, cardioprotective medications should be restarted before discharge [36]. During follow-up, SCr and urine protein should be determined and monitored in all AKI survivors at 1–12 weeks post-discharge depending on risk factors and degree of kidney recovery [38]. Finally, nephrology referral should be considered in patients with persistent poor kidney recovery and/or GFR ≤ 30 mL/min/1.73 m^2^ [39].

This is one of the first studies highlighting the high risk of CKD in VV-ECMO survivors. However, we acknowledge some limitations of our study. Firstly, this was a single-centre study and might not be representative of other ECMO centres. Patient-level criteria for ECMO initiation and ventilation settings before ECMO initiation are not available. However, ECMO was offered and delivered according to clear guidance from the NHS commissioning document, and the mortality rates are in keeping with the UK data [15] and remained similar over time (Additional file 1: Table S1). Second, we acknowledge that few patients had true baseline SCr values. We made every possible attempt to retrieve results from local care records or general practitioners and assume that the remaining patients had not had their kidney function checked in the 12 months prior to falling ill. This is supported by the fact that they were younger than average patients in the ICU and had only few comorbidities. It is likely that this group also had a low prevalence of CKD. Comparison of patient-centred outcomes including in-hospital and 1-year mortality also showed similar outcomes between using 3 baseline SCr methods. In addition, a subgroup analysis of patients with true baseline SCr values also corroborated with the results of the whole cohort. Less than half of the patients had SCr results available within 1 year of hospital discharge and thus the incidence of CKD may have been overestimated. We acknowledge that a proportion of these patients may have had SCr measurements which we were unable to retrieve. We also compared the characteristics of patients with and without available SCr results and found no significant difference. Third, the ESKD data were available from the UKRR until December 2017. It is possible that the risk of ESKD increased beyond 1 year. Fourth, the initiation of RRT was at clinicians’ discretion and could have impacted the different trajectories of kidney function. However, RRT was delivered by a team of highly skilled critical care nurses according to the departmental protocol. Fifth, although the study included all eligible patients in our centre over 6 years, the total number of patients eligible for analysis was 300 and some analyses were not possible such as the difference in outcomes between recovery and non-recovery AKI. Finally, we were unable to determine the exact causes of death. Nevertheless, this is the largest study which focuses on solely VV-ECMO adult patients and explored longer-term outcomes beyond hospital discharge. Also, we used both SCr and urine output criteria to diagnose AKI. In non-ECMO patients, it has been shown that patients meeting both urine output and SCr criteria are at increased risk of worse short-term and long-term outcomes compared with patients who meet the SCr criteria alone [40]. In our cohort, 23% of patients had AKI based on urine output criteria alone and would have been missed if we had only used SCr criteria. Whilst these findings should be validated externally with longer follow-up duration for kidney outcomes, our analysis highlights a significant gap in aftercare. Apart from a structured individualised follow-up care plan, there is a need for more research and intervention studies in post-AKI care. In particular, the role of biomarkers to predict patients at high-risk of developing worse kidney outcomes, the role of protective drugs like renin–angiotensin aldosterone inhibitors or statins in prevention of CKD after AKI, and the effectiveness of post-AKI clinic should be explored [41–43].

## Conclusion

Patients who received VV-ECMO in combination with RRT for AKI were at high risk of long-term mortality and CKD. Monitoring of kidney function was infrequent in routine clinical care post-hospitalisation. SCr results at discharge showed modest correlation with follow-up values, supporting the current recommendation to monitor kidney function after discharge. Clinicians and patients should be made aware of long-term kidney complications in this high-risk group. There is a need for appropriate follow-up and prevention strategies.

## Supplementary Information

**Additional file 1: Figure S1*.*** Study flowchart. **Figure S2:** Median (interquartile range) of serum creatinine values at discharge and at 6 and 12 months following discharge, in patients with all three measurements available (n = 51); p < 0.001, p = 0.104 and p = 0.146 for comparisons of creatinine at discharge vs. 6 months, discharge vs. 12 months and 6 months vs. 12 months, respectively. **Table S1.** Prevalence and outcomes of patients in the AKI-RRT, AKI-No RRT, and non-AKI groups, stratified by ECMO start year. **Table S2.** Relationship between ECMO start date and date of ICU admission. **Table S3.** Onset of AKI (n = 230) relative to ECMO start date. **Table S4.** Timing of RRT initiation relative to ECMO start date (n = 178). **Table S5.** Indications for RRT initiation by year of ECMO initiation. **Table S6.** 1-year mortality stratified by RRT status and date of RRT initiation relative to ECMO start date. **Table S7.** Adjusted hazard ratios of the association between RRT status relative to extracorporeal membrane oxygenation start date and overall mortality. **Table S8.** Characteristics of patients who survived to 1-year with and without SCr measurements available at 1-year. **Table S9.** AKI and RRT incidence, in-hospital mortality and 1-year mortality compared between using different baseline SCr methods. **Table S10.** Outcomes of cohort with true SCr results available.

**Additional file 2.** NHS ECMO referral criteria.

## Data Availability

The datasets used and/or analysed during the current study are available from the corresponding author on reasonable request.

## Abbreviations

AKI: Acute kidney injury
ARDS: Acute respiratory distress syndrome
CI: Confidence interval
CKD: Chronic kidney disease
CKD-EPI: Chronic kidney disease-epidemiology collaboration
COVID-19: Coronavirus disease-19
ECCO2R: Extracorporeal carbon dioxide removal
ECMO: Extracorporeal membrane oxygenation
ESKD: End-stage kidney disease
GFR: Glomerular filtration rate
GP: General practitioner
HR: Hazard ratio
ICU: Intensive care unit
IQR: Interquartile range
KDIGO: Kidney Diseases: Improving Global Outcomes
MDRD: Modification of diet in renal disease
NHS: National Health Service
RRT: Renal replacement therapy
SCr: Serum creatinine
SOFA: Sequential Organ Failure Assessment
UK: United Kingdom
UKRR: United Kingdom Renal Registry
VA-ECMO: Veno-arterial extracorporeal membrane oxygenation
VV-ECMO: Veno-venous extracorporeal membrane oxygenation
