# Supplementary material for: Long-term outcomes in patients who received veno-venous extracorporeal membrane oxygenation and renal replacement therapy: a retrospective cohort study
Source: Ann Intensive Care. 2022 Jul 23;12:70. doi: 10.1186/s13613-022-01046-0 (PMC9308118; doi:10.1186/s13613-022-01046-0)
Supplement: Supplementary file 1 — Additional file 1: Figure S1. Study flowchart. Figure S2: Median (interquartile range) of serum creatinine values at discharge and at 6 and 12 months following discharge, in patients with all three measurements available (n = 51); p < 0.001, p = 0.104 and p = 0.146 for comparisons of creatinine at discharge vs. 6 months, discharge vs. 12 months and 6 months vs. 12 months, respectively. Table S1. Prevalence and outcomes of patients in the AKI-RRT, AKI-No RRT, and non-AKI groups, stratified by ECMO start year. Table S2. Relationship between ECMO start date and date of ICU admission. Table S3. Onset of AKI (n = 230) relative to ECMO start date. Table S4. Timing of RRT initiation relative to ECMO start date (n = 178). Table S5. Indications for RRT initiation by year of ECMO initiation. Table S6. 1-year mortality stratified by RRT status and date of RRT initiation relative to ECMO start date. Table S7. Adjusted hazard ratios of the association between RRT status relative to extracorporeal membrane oxygenation start date and overall mortality. Table S8. Characteristics of patients who survived to 1-year with and without SCr measurements available at 1-year. Table S9. AKI and RRT incidence, in-hospital mortality and 1-year mortality compared between using different baseline SCr methods. Table S10. Outcomes of cohort with true SCr results available. [file 13613_2022_1046_MOESM1_ESM.docx]

**Additional File 1**

**Figure S1: Study flowchart**


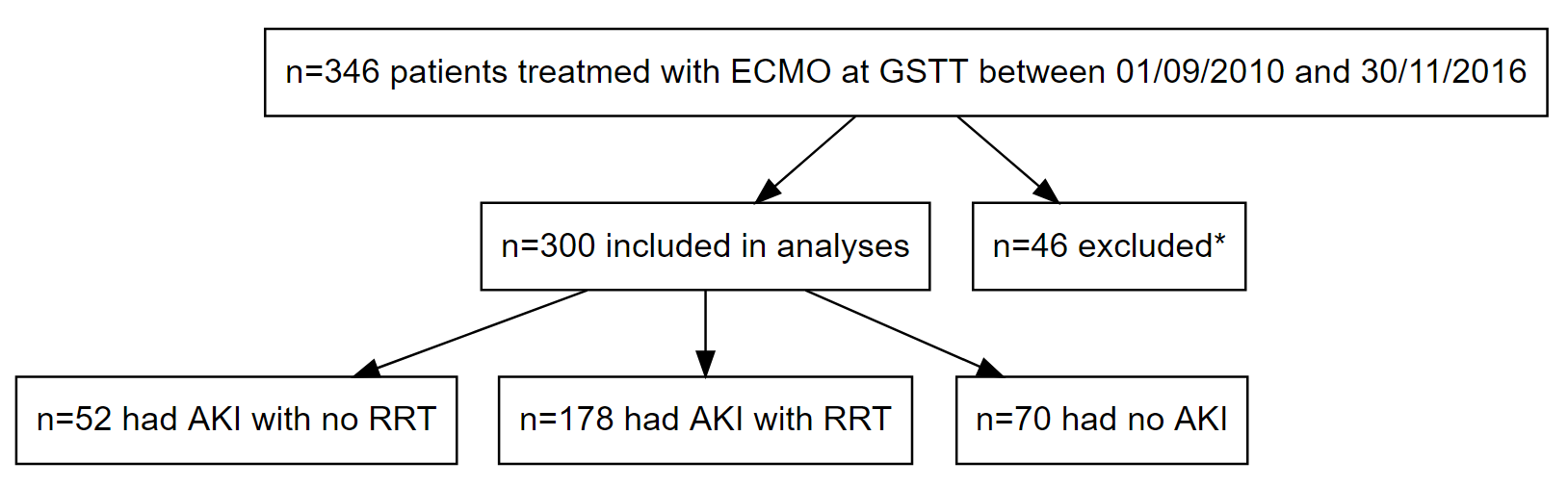


*Patients were excluded if they were < 18 years old (n=5), had existing ESRD or KT (n=8), survival after ECMO initiation for < 48 hours (n=15) or received veno-arterial ECMO or ECCO2R (n=18)

**Abbreviations:** ECMO, extracorporeal membrane oxygenation; ECCO2R, extracorporeal carbon dioxide removal; ESRD, end-stage renal disease; KT, kidney transplantation

**Figure S2: Median (interquartile range) of serum creatinine values at discharge and at 6 and 12 months following discharge, in patients with all three measurements available (n=51); p<0.001, p=0.104 and p=0.146 for comparisons of creatinine at discharge vs 6 months, discharge vs 12 months and 6 months vs 12 months, respectively.**

**
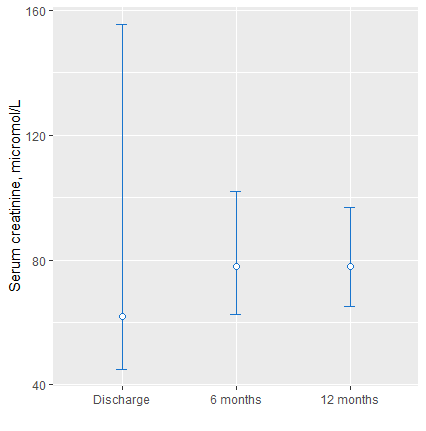
**

**Table S1: Prevalence and outcomes of patients in the AKI-RRT, AKI-No RRT, and non-AKI groups, stratified by ECMO start year**

| **ECMO start year** | **N** | **AKI, RRT** | **AKI, No RRT** | **No AKI** | **ECMO duration (days)** | **In-hospital mortality** | **1 year mortality** | **ESKD at 12 months** |
| --- | --- | --- | --- | --- | --- | --- | --- | --- |
| Sept – Dec 2010 | 2 | 1 (50.0%) | 1 (50.0%) | 0 (0.0%) | 19.5 (18, 21) | 1 (50.0%) | 1 (50.0%) | 0 (0.0%) |
| 2011 | 9 | 4 (44.4%) | 2 (22.2%) | 3 (33.3%) | 10 (8, 16) | 2 (22.2%) | 2 (22.2%) | 1 (11.1%) |
| 2012 | 34 | 19 (55.9%) | 3 (8.8%) | 12 (35.3%) | 9 (8, 13) | 9 (26.5%) | 10 (29.4%) | 0 (0.0%) |
| 2013 | 54 | 30 (55.6%) | 12 (22.2%) | 12 (22.2%) | 10 (6, 16) | 12 (22.2%) | 16 (29.6%) | 0 (0.0%) |
| 2014 | 65 | 38 (58.5%) | 12 (18.5%) | 15 (23.1%) | 10 (6, 14) | 16 (24.6%) | 18 (27.7%) | 0 (0.0%) |
| 2015 | 67 | 44 (65.7%) | 9 (13.4%) | 14 (20.9%) | 7 (5, 14) | 13 (19.4%) | 15 (22.4%) | 2 (3.0%) |
| 2016 | 69 | 42 (60.9%) | 13 (18.8%) | 14 (20.3%) | 9 (7,17) | 12 (17.4%) | 17 (24.6%) | 0 (0.0%) |
| Overall | 300 | 178 (59.3%) | 52 (17.3%) | 70 (23.3%) | 9.0 (6.0, 16.0) | 65 (21.7%) | 79 (26.3%) | 3 (1.0%) |

**Abbreviation:** ECMO, extracorporeal membrane oxygenation; AKI, acute kidney injury; RRT, renal replacement therapy; ESKD, end-stage kidney disease

**Table S2: Relationship between ECMO start date and date of ICU admission**

| **Days from ICU admission to ECMO start date** | **Number of patients (%)** |
| --- | --- |
| 0 | 123 (41) |
| 1 | 93 (31) |
| 2 | 23 (7.7) |
| 3 | 6 (2) |
| 4 | 11 (3.7) |
| 5 | 7 (2.3) |
| 6-10 | 19 (6.3) |
| 11-20 | 16 (5.3) |
| 21+ | 2 (0.7) |
| Total | 300 |

**Abbreviations:** ICU, intensive care unit; ECMO, extracorporeal membrane oxygenation

**Table S3: Onset of AKI (n=230) relative to ECMO start date**

| **Timing of AKI diagnosis** | **AKI, RRT** | **AKI, no RRT** | **Number (%)** |
| --- | --- | --- | --- |
| Before ECMO start date | 52 (29.2) | 7 (13.5) | 59 (25.6) |
| On ECMO start date | 97 (54.5) | 24 (46.2) | 121 (52.6) |
| 1-3 days after ECMO start date | 18 (10.1) | 6 (11.5) | 24 (10.4) |
| 4-6 days after ECMO start date | 3 (1.7) | 10 (19.2) | 13 (5.7) |
| 7-14 days after ECMO start date | 6 (3.4) | 5 (9.6) | 11 (4.8) |
| 15-17 days after ECMO start date | 2 (1.1) | 0 | 2 (0.9) |
| Total | 178 | 52 | 230 |

**Abbreviations:** AKI, acute kidney injury; ECMO, extracorporeal membrane oxygenation; RRT, renal replacement therapy

**Table S4: Timing of RRT initiation relative to ECMO start date (n=178)**

| **Timing of RRT initiation** | **Number (%)** |
| --- | --- |
| Before ECMO start date | 31 (17.4) |
| On ECMO start date | 88 (49.4) |
| 1-3 days after ECMO start date | 28 (15.7) |
| 4-6 days after ECMO start date | 10 (5.6) |
| 7-14 days after ECMO start date | 13 (7.3) |
| 15-37 days after ECMO start date | 8 (4.5) |
| Total | 178 |

**Abbreviations:** ECMO, extracorporeal membrane oxygenation; RRT, renal replacement therapy

**Table S5: Indications for RRT initiation by year of ECMO initiation**

| **Year** | **VV ECMO patients** | **Patients receiving RRT** | **K** | **Acidosis** | **Volume control/fluid overload** | **Persistent Oliguria** | **Uraemia** | **Others** |
| --- | --- | --- | --- | --- | --- | --- | --- | --- |
|  |  |  | **Percentage by RRT** | | | | | |
| Sept – Dec 2010 | 2 | 1 | 0 | 0 | 1 (100%) | 0 | 0 | 0 |
| 2011 | 9 | 4 | 0 | 0 | 3 (75%) | 1 (25%) | 1 (25%) | 1 (25%) |
| 2012 | 34 | 19 | 1 (5.3%) | 3 (15.8%) | 8 (42.1%) | 8 (42.1%) | 6 (31.5%) | 4 (21.1%) |
| 2013 | 54 | 30 | 2 (6.6%) | 8 (26.6%) | 10 (33.3%) | 8 (26.6%) | 5 (16.7%) | 3 (10%) |
| 2014 | 65 | 38 | 2 (5.2%) | 13 (34.2%) | 13 (34.2%) | 13 (34.2%) | 7 (18.4%) | 7 (18.4%) |
| 2015 | 67 | 44 | 3 (6.8%) | 10 (22.7%) | 15 (34%) | 17 (38.6%) | 10 (22.7%) | 5 (11.3%) |
| 2016 | 69 | 42 | 4 (9.5%) | 4 (9.5%) | 22 (52.3%) | 8 (19%) | 10 (23.8%) | 12 (28.5%) |
| Total | 300 | 178 | 12 (6.8%) | 38 (21.6%) | 72 (40.9%) | 55 (31.3%) | 39 (22.2%) | 32 (18.2%) |

* 62 patients had ≥2 indications

** Other indications were sodium control and rhabdomyolysis.

**Table S6: 1-year mortality stratified by RRT status and date of RRT initiation relative to ECMO start date**

| **Timing of RRT initiation** | **N** | **Died** |
| --- | --- | --- |
| No RRT | 122 | 22 (18.0%) |
| RRT |  |  |
| - Before ECMO start date | 31 | 7 (22.6%) |
| - On ECMO start date | 88 | 31 (35.2%) |
| - After ECMO start date | 59 | 19 (32.2%) |
| Total | 300 | 300 (26.3%) |

**Abbreviations:** ECMO, extracorporeal membrane oxygenation; RRT, renal replacement therapy

**Table S7: Adjusted hazard ratios of the association between RRT status relative to extracorporeal membrane oxygenation start date and overall mortality**

| **Characteristic** | **Unadjusted HR** | **95% CI** | **P-value** | **Adjusted HR** | **95% CI** | **P-value** |
| --- | --- | --- | --- | --- | --- | --- |
| **RRT group** |  |  |  |  |  |  |
| No RRT | 1 (ref) | - | - | 1 (ref) | - | - |
| RRT before ECMO start date | **1.66** | **1.07 to 2.57** | **0.023** | 1.27 | 0.79 to 2.02 | 0.327 |
| RRT same day or after ECMO start date | **1.79** | **1.04 to 3.05** | **0.034** | 1.37 | 0.78 to 2.38 | 0.271 |
| **Age (per 5 years)** | 4.46 | 1.24, 17.9 | 0.023 | 1.11 | 1.03 to 1.19 | 0.004 |
| **Chronic liver disease** | 11.8 | 3.55, 53.1 | <0.001 | 4.02 | 1.81 to 8.92 | <0.001 |
| **Any malignancy** | 0.54 | 0.31, 0.92 | 0.023 | 7.76 | 4.29 to 14.0 | <0.001 |
| **Lowest albumin on admission (per 10 g/L)** | 4.46 | 1.24, 17.9 | 0.023 | 0.68 | 0.43 to 1.09 | 0.108 |

**Abbreviations:** RRT, renal replacement therapy; ECMO, extracorporeal membrane oxygenation; HR, hazard ratio; CI, confidence interval

**Table S8: Characteristics of patients who survived to 1-year with and without SCr measurements available at 1-year**

| **Characteristic^1^** | **SCr available,**  **n = 91** | **SCr unavailable,**  **n = 130** | **p-value^2^** |
| --- | --- | --- | --- |
| **AKI / RRT status during hospitalization** |  |  | 0.29 |
| AKI, RRT | 54 (59.3%) | 67 (51.5%) |  |
| AKI, No RRT | 14 (15.4%) | 31 (23.8%) |  |
| No AKI | 23 (25.3%) | 32 (24.6%) |  |
| **SCr at hospital admission** | 104.0 (66.5, 191.0) | 128.5 (73.0, 222.2) | 0.20 |
| **GFR at hospital admission** | 56.0 (31.5, 103.5) | 49.0 (25.0, 94.8) | 0.40 |
| **SCr at discharge** | 70.0 (46.5, 169.0) | 73.0 (51.0, 146.8) | 0.31 |
| **GFR at discharge** | 95.0 (35.5, 120.0) | 90.5 (41.5, 120.0) | 0.88 |
| **SCr at 6 months** | 78.0 (61.0, 103.0) | NA | NA |
| **GFR at 6 months** | 80.0 (67.0, 103.0) | NA | NA |
| **SCr at 1 year** | 79.0 (64.0, 102.5) | NA | NA |
| **GFR at 1 year** | 81.9 (67.0, 98.5) | NA | NA |
| **Male** | 50 (54.9%) | 76 (58.5%) | 0.68 |
| **Ethnicity^3^** |  |  | 0.13 |
| White | 64 (71.9%) | 108 (83.7%) |  |
| Black | 10 (11.2%) | 8 (6.2%) |  |
| Asian | 12 (13.5%) | 8 (6.2%) |  |
| Mixed | 0 (0.0%) | 0 (0.0%) |  |
| Other/not stated | 3 (3.4%) | 5 (3.9%) |  |
| **Age (years)** | 44.0 (32.0, 53.0) | 43.0 (34.2, 53.0) | 0.98 |
| **Weight (kg)** | 84.0 (70.0, 101.0) | 80.0 (67.8, 101.0) | >0.99 |
| **BMI (kg/m^2^)** | 27.9 (24.4, 33.6) | 26.5 (23.9, 33.7) | 0.52 |
| **Diabetes** | 11 (12.1%) | 13 (10.0%) | 0.66 |
| **Hypertension** | 16 (17.6%) | 20 (15.4%) | 0.71 |
| **Congestive heart failure** | 3 (3.3%) | 4 (3.1%) | >0.99 |
| **Coronary artery disease** | 1 (1.1%) | 4 (3.1%) | 0.65 |
| **Atrial fibrillation** | 1 (1.1%) | 1 (0.8%) | >0.99 |
| **Peripheral artery disease** | 1 (1.1%) | 0 (0.0%) | 0.41 |
| **Cerebrovascular accidents** | 3 (3.3%) | 1 (0.8%) | 0.31 |
| **Chronic lung disease** | 21 (23.1%) | 35 (26.9%) | 0.53 |
| **Chronic liver disease** |  |  | >0.99 |
| No | 89 (97.8%) | 128 (98.5%) |  |
| Mild to moderate liver disease | 2 (2.2%) | 2 (1.5%) |  |
| Severe liver disease | 0 (0.0%) | 0 (0.0%) |  |
| **Any active malignancy within 5 years** | 3 (3.3%) | 0 (0.0%) | 0.068 |
| **Type of malignancy** |  |  | 0.068 |
| None | 88 (96.7%) | 130 (100.0%) |  |
| Solid tumour | 1 (1.1%) | 0 (0.0%) |  |
| Hematologic tumour | 2 (2.2%) | 0 (0.0%) |  |
| **Other immunosuppressive conditions** | 10 (11.0%) | 6 (4.6%) | 0.11 |
| **Chronic kidney disease** | 2 (2.2%) | 4 (3.1%) | >0.99 |
| **Use of nephrotoxic drugs** | 23 (25.3%) | 34 (26.2%) | >0.99 |
| ^1^ Statistics presented: Median (interquartile range); n (%) | | | |
| ^2^ Statistical tests performed: t-test; Fisher's exact test | | | |
| ^3^ Missing data: eGFR at 6 months (N=14), Ethnicity (N=3), Weight (N=12), BMI (N=34) | | | |

**Abbreviations:** AKI, acute kidney injury; BMI, body mass index; GFR, glomerular filtration; NA, not applicable; RRT, renal replacement therapy; SCr, serum creatinine

**Table S9: AKI and RRT incidence, in-hospital mortality and 1-year mortality compared between using different baseline SCr methods**

| Outcomes | Total | Cohort with true baseline SCr (n=31) | Cohort with first hospital admission SCr used as baseline SCr (n=88) | Cohort with SCr estimated by back calculation (n=177) |
| --- | --- | --- | --- | --- |
| Baseline SCr (umol/L) (median, IQR) | 80 (69, 97) | 73 (60, 87) | 60 (47, 72) | 95 (79, 100) |
| AKI incidence in ICU | 76.7% | 27 (81.8%) | 32 (36%) | 171 (96.1%) |
| RRT incidence whilst in ICU | 59.3% | 23 (69.7%) | 14 (15.7%) | 141 (79.2%) |
| In-hospital mortality | 21.7% | 5 (15.2%) | 14 (15.7%) | 46 (25.8%) |
| 1-year mortality | 26.3% | 6 (18.2%) | 20 (22.5%) | 53 (29.8%) |

**Abbreviations:** AKI, acute kidney injury; ICU, intensive care unit; RRT, renal replacement therapy; SCr, serum creatinine; IQR, interquartile range

**Table S10: Outcomes of cohort with true SCr results available**

| **Characteristic** | **Overall**  **n = 33** | **AKI, RRT**  **n = 23** | **AKI, no RRT**  **n = 4** | **No AKI**  **n = 6** |
| --- | --- | --- | --- | --- |
| **1-year mortality** | 6 (18.2%) | 5 (21.7%) | 0 | 1 (16.7%) |
| **(Survivors)** | 27 | 18 | 4 | 5 |
| **End-Stage kidney disease** | 3 | 3 | 0 | 0 |
| **SCr available at 1 year in survivors** | 21 (77.8%) | 12 (66.7%) | 4 (100%) | 5 (100%) |
| **Chronic kidney disease** | 5 (23.8%) | 5 (41.7%) | 0 | 0 |
| **SCr values at 6 months (mmol/L)** (n=18) | 80 (65, 102) | 88 (54, 122) | 82 (65, 87) | 78 (71, 94) |
| **SCr values at 12 months (mmol/L)**  (n=18) | 77 (66, 97) | 97 (46, 109) | 77 (67, 80) | 74 (67, 80) |
| **SCr values at 12 months or 6 months if unavailable (mmol/L)** (n=22) | 78 (66, 102) | 97 (46, 122) | 77 (67, 80) | 74 (67, 80) |

**Abbreviations:** AKI, acute kidney injury; ICU, intensive care unit; RRT, renal replacement therapy; SCr, serum creatinine; IQR, interquartile range
