# Supplementary material for: Long-term outcomes in patients who received veno-venous extracorporeal membrane oxygenation and renal replacement therapy: a retrospective cohort study
Source: Ann Intensive Care. 2022 Jul 23;12:70. doi: 10.1186/s13613-022-01046-0 (PMC9308118; doi:10.1186/s13613-022-01046-0)
Supplement: Supplementary file 2 — Additional file 2. NHS ECMO referral criteria. [file 13613_2022_1046_MOESM2_ESM.docx]

**Additional File 2**

**NHS ECMO referral criteria**

**Inclusion criteria**

- Patients with demonstrable severe respiratory failure from a non-cardiac cause (i.e. Murray Lung Injury score 3.0 or above, or uncompensated hypercapnia with a pH < 7.20 despite optimal conventional treatment);
- Patients for whom ongoing positive pressure ventilation is not appropriate (e.g. significant tracheal injury)

**Exclusion criteria**

- Contraindication to continuation of active treatment (e.g. unsurvivable extrapulmonary disease)
- Severe life-limiting comorbidity such that ECMO support is unlikely to result in survival with quality of life (e.g. advanced malignancy, severe immunocompromise)
- Any clinical feature likely to lead to dependency on ECMO and inability to wean (e.g. profound muscle weakness, significant irreversible pulmonary fibrosis caused by either underlying disease or duration of mechanical ventilation).

**Reference:** https://www.england.nhs.uk/wp-content/uploads/2019/02/Adult-ECMO-Service-Specification.pdf
